# A Sporadic Four-Year Hospital Outbreak of a ST97-IVa MRSA With Half of the Patients First Identified in the Community

**DOI:** 10.3389/fmicb.2018.01494

**Published:** 2018-07-10

**Authors:** Ingrid M. Rubin, Thomas A. Hansen, Anne Mette Klingenberg, Andreas M. Petersen, Peder Worning, Henrik Westh, Mette D. Bartels

**Affiliations:** ^1^Department of Clinical Microbiology, Hvidovre Hospital, Hvidovre, Denmark; ^2^Department of Gastroenterology, Hvidovre Hospital, Hvidovre, Denmark; ^3^Institute of Clinical Medicine, University of Copenhagen, Copenhagen, Denmark

**Keywords:** WGS, outbreak, CO-MRSA, ST97, HCWs

## Abstract

This study describes a sporadically occurring 4-year outbreak of methicillin-resistant *Staphylococcus aureus* (MRSA) originating from a surgical ward. Whole-genome sequencing (WGS) identified the outbreak clone as *spa* type t267, sequence type ST97, and SCC*mec* IVa. Prompted by the finding of four patients within 6 months in the same ward with this unusual MRSA type, an outbreak was suspected. Subsequent MRSA screening in the ward in February 2017 identified three-additional patients and two health care workers (HCWs) with t267/ST97-IVa. WGS linked these 9 isolates to 16 previous isolates in our WGS database and the outbreak thus included 23 patients and two HCWs. Twenty-one patients had a connection to the surgery ward during the period 2013–2017, but half of them had MRSA diagnosed in the community long after discharge. The community debut of several patients MRSA infections weeks to months after hospital discharge made the identification of a hospital source difficult and it was the SNP relatedness of the isolates that led us to identify the common denominator of hospitalization. An index patient was not identified, but our hypothesis is that HCWs with unrecognized long-term MRSA colonization could have caused sporadic nosocomial transmission due to intermittent breaches in infection prevention and control practice.

## Introduction

Methicillin-resistant *Staphylococcus aureus* (MRSA) has been a global medical challenge since its emergence in 1961, 2 years after methicillin was introduced to treat penicillin-resistant *S. aureus* (Jevons, 1961). In human medicine, the focus has traditionally been on the hospital-acquired clones (HA-MRSA), but new clones have emerged both in livestock and in community settings (DeLeo et al., 2010; Gonçalves da Silva et al., 2017). Outbreaks in hospitals and nursing homes are today caused by both HA-MRSA and community-associated MRSA (CA-MRSA) clones (DeLeo et al., 2010; Di Ruscio et al., 2017; Henderson and Nimmo, 2017) and it has been suggested no longer to regard HA-MRSA and CA-MRSA as separate entities (Zarfel et al., 2013). Hospital admission of unknown MRSA carriers, lack of MRSA admittance screening and spread of MRSA among nursing home residents could all be part of the explanation of this mélange of HA-MRSA and CA-MRSA in hospitals (Gonzalez et al., 2006). General contact procedures, an important part of infection control, will keep patients safe from transmission of MRSA from health care workers (HCWs) (Harris A.D. et al., 2013, Harris et al., 2017). By typing MRSA isolates and drawing interferences on transmission based on genetic relatedness, transmission pathways can be tracked (Harris S.R. et al., 2013). Today the most precise typing of bacteria is by whole-genome sequencing (WGS). WGS has proven an excellent tool, with good inter-laboratory reproducibility in hospital outbreaks (Bartels et al., 2013; Leopold et al., 2014; SenGupta et al., 2014).

Here, we describe a prolonged outbreak of MRSA initiated in a hospital ward that was confirmed by WGS. Tracing of the clone led us to 23 patients and two HCWs, who in most cases had a common denominator in one of the hospitals surgery wards.

## Materials and Methods

### Setting

This retrospective study analyzed an outbreak spanning the period between June 2013 and February 2017 that occurred at Hvidovre Hospital, Copenhagen, Denmark. The MRSA isolates studied were routinely found in clinical samples or after the outbreak was discovered in February 2017 through MRSA screening of patients and staff.

### Data Set

All MRSA isolates are investigated by WGS as part of the routine at the Department of Clinical Microbiology, Hvidovre Hospital. In order to define the outbreak, we investigated the relatedness to other t267/ST97/SCC*mec* IVa isolates from the period 2013–2017 in the Capital Region of Denmark and these isolates were included in the SNP analysis.

### Whole-Genome Sequencing and Analysis

Each MRSA isolate was initially confirmed with an in-house multiplex real-time polymerase chain reaction (PCR) that detects the presence of *nuc*, *fem*A, *mec*A, and *mec*C. Since January 2013 all MRSA isolates have been WGS on a MiSeq (Illumina, United States). DNA extraction were performed on all MRSA isolates and libraries were made with 2 × 150 bp paired-end Nextera XT DNA sample preparation kit (Illumina, United States) and sequenced on a MiSeq (Illumina, United States). The reads were mapped to a USA300 reference sequence (US300_TCH1516) using stampy (Lunter and Goodson, 2011) with an expected substitution rate of 0.01 (Didelot et al., 2012) for single nucleotide variants detection. Variants were called using SAMtools v0.1.12 (Li, 2011) mpileup command with options -M0 -Q30 -q30 -o40 -e20 -h100 -m2 -D -S. The genome was assembled using Velvet v1.0.11 (Zerbino and Birney, 2008) or Spades (Bankevich et al., 2012). Phylogeny was inferred by neighbor-joining analysis.

### Ethical Considerations

Permission to link the sequencing of MRSA from routine clinical samples to patient data without individual patient consent was obtained from the Danish Data Protection Agency (no. AHH-2017-095, I-Suite nr. 06029). Permission to look up the patients admission data without individual consent was obtained from the Hospital Board (no. WZ17038300-2018-14).

## Results

### Documentation of the Outbreak

In January 2017, an MRSA infected patient was found in one of the hospital’s surgery wards. From the sample of abdominal pus two distinct MRSA types were found based on antimicrobial susceptibility patterns. WGS of the two isolates identified a t267/ST97-IVa and a t002/ST5-IVg. Looking 6 months back in our MRSA WGS database, we identified three other patients admitted to the same ward that had tested positive for MRSA t267/ST97. Searching our WGS database back to 2013 we found this to be an unusual MRSA type in our region with just 34 isolates from 2013 to January 2017 (0.7%). A neighbor-joining tree was then constructed with all 34 isolates showing 20 isolates with a close connection of ≤50 SNPs. These isolates also shared the same SCC*mec* type, namely IVa. Thus, the suspicion of an MRSA outbreak was confirmed. This led to a several week-long screening of patients, HCWs and other staff members in the ward resulting in the finding of three-additional patients and two HCWs with t267/ST97-IVa. Furthermore, screening at the ward revealed that two HCWs carried other MRSA types (t045/ST5-IVc/e, t002/ST5-IVg) and one patient with known contact to pigs carried the livestock-associated t034/ST398-V.

**FIGURE 1:**
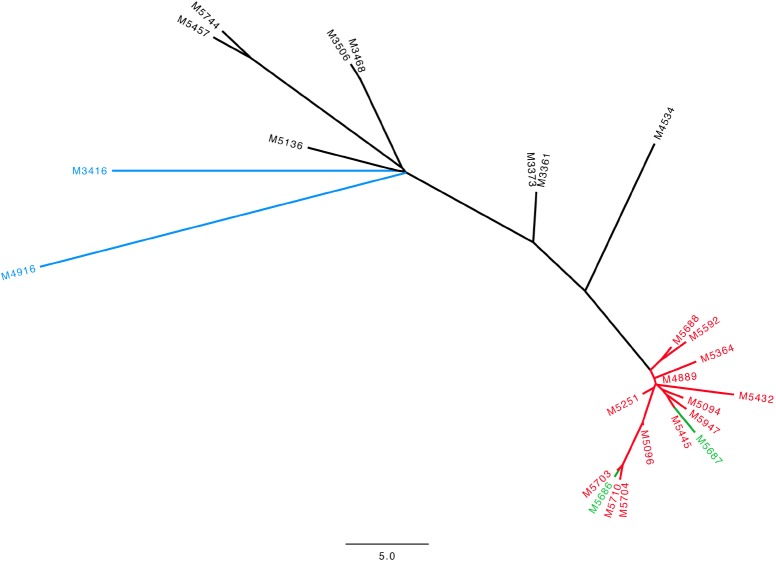
Neighbor-joining tree for the 25 isolates in our outbreak cluster with t267/ST97 SCC*mec* IVa. Scale bar indicates the SNP distance. Green: HCWs, Red: patients admitted in 2016 and 2017, Black: patients admitted 2013–2015, Blue: patients with no identified relation to the surgical ward.

In our final outbreak cluster (**Figure 1**) we ended up with 25 persons. Eighteen had been admitted to the surgical ward, two were HCWs, two were family members of MRSA positive patients, and one had shared a room at another ward in another hospital with an MRSA positive patient, who had previously been admitted to the surgery ward. Finally, two patients had no relation either to another patient or to the ward to the best of our knowledge. We have data on hospitalization at the surgery ward for most patients but the surgery ward has many sub-departments and we lack data on room allocations as well as the exact department admitted to. Our data show that 13 patients had an overlap in admission period with at least one other patient (**Supplementary Figure S1**).

Nine patients had been diagnosed with MRSA t267/ST97-IVa at their General Practitioner and three more had been diagnosed at the Emergency Room, resulting in 12 cases first identified in the community, here defined as community-onset MRSA (CO-MRSA). Eight patients had their MRSA diagnosed at hospital wards HA-MRSA. Five persons had been diagnosed by screening of the hospital ward (including the two HCWs) (**Table 1**).

**Table 1:** Demographics.

Patients	Time of MRSA positive	Year admitted to the surgery ward	Sites of colonization	Hospital-acquired vs. community-onset	Comment
M5592	January 2017	2017	Abdominal pus	HA	
M5703	February 2017	2017	Screening ward	HA	Shared room with M5704
M5704	February 2017	2017	Screening ward	HA	Shared room with M5703
M5688	February 2017	2017	Screening ward	HA	
M5710	February 2017	2016 and 2017	Wound	CO	
M5744	March 2017	2016	Tracheal secretion	CO	
M5445	November 2016	2013 and 2016	Cicatrice	HA	
M5432	November 2016	2016	Urine	CO	
M5251	September 2016	2016	Catheter	HA	
M5136	July 2016	2016	Urine	HA	
M5096	July 2016	2016	Blood	HA	
M5094	June 2016	2016	Absces	CO	
M4889	March 2016	2016	Cicatrice	CO	
M4534	September 2015	2015 and 2016	Cicatrice	CO	
M5947	June 2017	2015 and 2016	Wound	CO	
M5364	October 2016	2015	Cicatrice	CO	
M3506	March 2014		Screening	HA	Screening due to MRSA positive household contact (M3468)
M3468	February 2014	2014	Wound	CO	Household contact with M3506
M3361	December 2013	2013	Nose	HA	
M5687	February 2017		Screening ward		HCW
M5686	February 2017		Screening ward		HCW
M5457	November 2016		Screening	CO	Screening due to MRSA positive household contact (M4744)
M3373	December 2013		Screening	HA	Shared room at another hospital with ++ M3361 in 2013
M4916	April 2016		Screening GP	CO	Unknown connection
M3416	January 2014		Screening GP	CO	Unknown connection

Due to the finding of other MRSA types among our HCWs, we also studied the phylogenetic tree of all the t045/ST5-IVc/e and t002/ST5-IVg isolates in our WGS database. There were only three t045/ST5 isolates and the two others had another SCC*mec* type (**Supplementary Figure S2**). Our database contained 266 t002/ST5 isolates of which 40 harbored SCC*mec* IVg (**Supplementary Figure S3**).

### SNP Analysis and Characterization of the Outbreak Clone

The 25 t267/ST97/IVa isolates showed a low diversity with a maximum of 50 SNP differences over the 4-year period. Within our outbreak cluster, a further sub-cluster could be distinguished with 15 isolates with a maximum of 11 SNPs. This sub-cluster consisted of 13 patients admitted in 2016 and 2017 as well as the two HCWs.

All outbreak isolates belonged to *spa* type t267 and ST97 except for two isolates with an unnamed ST that was a single locus variant (SLV) of ST97. All isolates had SCC*mec* IVa and no isolates had Panton–Valentine leukocidin (PVL) or the arginine catabolic mobile element (ACME). The 14 non-outbreak related isolates of t267/ST97 had SCC*mec* IVc/e (Gonzalez et al., 2006), SCC*mec* IVa (Henderson and Nimmo, 2017), and SCC*mec* V (Gonçalves da Silva et al., 2017). The outbreak isolates were resistant to methicillin and susceptible to erythromycin, clindamycin, gentamicin, fusidic acid, linezolid, mupirocin, trimethoprim/sulfamethoxazole, and rifampicin.

### Infection Control Measures

An outbreak group with representatives from the surgical ward, the Department of Cleaning, the Department of Clinical Microbiology, and the Infection Control Organization was established. In order to exclude shortcomings of general infection control precautions, various initiatives were launched. The ward had daily visits by the infection control and prevention nurse, where behavior was observed and adherence to procedures monitored, and on this basis an increased focus was placed on the use of protective equipment such as gloves and plastic aprons, which among other things led to an increased availability of protective equipment in the department. Furthermore, focus was on infection control precautions for both HCWs and patients and in particular on how patients could be motivated for better hand hygiene.

The ward was cleaned by standard hospital cleaning, followed by manual disinfection with bleach. The HCWs with the outbreak clone were successfully decolonized.

## Discussion

It is a global trend that patients are hospitalized for increasingly shorter time periods. Therefore, hospital acquisition of an MRSA might not be suspected or identified due to clinical onset long after discharge. Healthcare-associated MRSA outbreaks are rare in Denmark (Andersen and Knudse, 2016) and have been predominantly associated with outbreaks in Neonatal Wards (Ramsing et al., 2013; Bartels et al., 2015; Franck et al., 2017). The extent to which the community serves as a reservoir to the spread of MRSA into hospitals is largely unknown, but globally several reports on CA-MRSA in hospitals have emerged (DeLeo et al., 2010; Thurlow et al., 2012). With repeated introduction of CA-MRSA into hospitals (Cho and Chung, 2017; Coll et al., 2017), a better action plan is needed to tackle and curb community-associated carriage (Bartels et al., 2010). If the level of MRSA carriage increases in the general population then it will also increase in patients and HCWs. In this situation the sporadic spread of MRSA between HCWs and patients with unknown carrier-state, that we find in low MRSA prevalence countries might contribute to more intermittent transmission despite general infection control procedures.

Here, we report a 4-year long outbreak of MRSA type t267/ST97 SCC*mec* IVa, that was discovered as the MRSA was found in four patients in the same ward within 6 months. This, for us, rare MRSA has sporadically been found around the world and has been described both as a CA-MRSA (Monecke et al., 2011) and as a LA-MRSA in pigs and associated with bovine mastitis (Menegotto et al., 2012; Pantosti, 2012; Feltrin et al., 2015). We have routinely WGS all MRSA isolates since January 2013 and a phylogenetic tree clustered 25 isolates together and patient records revealed that the common denominator was the surgery ward. The outbreak isolates differed up to 50 SNPs, with a subcluster of 13 patients from 2016 or 2017 and the two HCWs whose isolates differed by no more than 11 SNPs. This gives an evolution of the core genome of about 5–6 SNPS per year in our sub-cluster comparable to the 6–9 SNPs per year described in one study (Holden et al., 2013) and more than the 3–4 SNPs per year in other studies (Harris et al., 2010; Senn et al., 2016). The remaining 14 isolates with the same MRSA type had no link to the ward in question and the SNP distance to the outbreak isolates was between 50 and 249 SNPs. Furthermore, most of these isolates had a different SCC*mec*, which also indicates another clone.

Since the outbreak was ongoing for such a prolonged period at one of our busiest surgical wards, the actual number of people infected or colonized with the MRSA clonal isolate is probably higher than the rather modest number we report. Only individuals in the community who had a clinical infection would have been identified through a sample so MRSA carriers can very well have eluded the system. There could be various explanations to the increase in the number of positive MRSA patients in 2016–2017 in this study. Of course, we found quite a few through the screening in 2017 that might not have been found if the screening had not been performed. Another aspect could be that there were unknown MRSA positive HCWs that had quit the ward before the screening in 2017, but contributed to the increase in cases in 2016 and 2017. Nevertheless, the finding of relatively few patients over a 4-year period indicates there is relatively little spread between HCWs and patients. Previous studies have concluded that nosocomial outbreaks caused by HCWs represent rare events, and therefore screening of personnel should not be performed regularly (Danzmann et al., 2013). Another study proposes three possible scenarios on the role of HCWs: being vectors of transmission, persistent reservoirs, or innocent by-standers, and concludes by suggesting aggressive screening and eradication policies in outbreak investigations (Albrich and Harbarth, 2008). Another aspect of the role of HCWs, and one that is enhanced by our findings, is that due to good infection control measures, HCWs with unrecognized long-term colonization cause only sporadic transmission. This is further supported by the finding of two other MRSA types in the HCWs during the ward screening, with no documented outbreaks caused by them. In support of the conclusion that there had been bacterial transmission from HCWs to patients is the fact that after the HCWs were declared free of MRSA, no more outbreak isolates have been seen at the ward as of May 2018. Of course, transmission could also have occurred between patients, since 13 patients had an overlap in admission time with at least one other patient. However, we lack data on room and sub-department allocation to support this. Due to good infection control measurements transmission only occurred rarely as the outbreak was going on unnoticed.

To automatically detect potential outbreaks the combination of detailed epidemiological data together with WGS is crucial (Roer et al., 2017). In the future an electronic system linking a patients isolate to the last seen most related isolate, will enable earlier detection of outbreaks (Mellmann et al., 2016).

## Conclusion

Whole-genome sequencing can enhance the detection of prolonged hospital outbreaks of MRSA. Furthermore, we bring to light the fact that increasingly shorter hospital stays delays outbreak detection if continuous analysis of WGS is not performed. In this study, we identified an MRSA outbreak in February 2017 and with the use of WGS we could trace the outbreak 4 years back in time.

We hypothesize that HCWs with an unknown MRSA carrier-state might cause sporadic transmission and sustain an unknown outbreak over many years. However, we believe that HCWs with a known MRSA carrier-state usually do not cause transmission due to their personally increased awareness of the importance of infection control standard precautions.

## Author Contributions

TH and PW performed the bioinformatics analyses. All authors contributed to the writing of the manuscript.

## Conflict of Interest Statement

The authors declare that the research was conducted in the absence of any commercial or financial relationships that could be construed as a potential conflict of interest.

## Supplementary Material

FIGURE S1 Timeline of hospitalization dates at the surgery ward and date of first positive MRSA sample. Thirteen patients had an overlap with at least one other patient. M5136 had an admission in June 2016, but the exact dates were not available. Sample date denotes the date when MRSA was first discovered in the patient.

FIGURE S2 Neighbor-joining tree for the three isolates with t045/ST5-IVc/e. Scale bar indicates the SNP distance. M5696 is the HCW. The difference between the isolates was more than 200 SNPs.

FIGURE S3 NJ-tree of the t002/ST5-IVg isolates. Scale bar indicates the SNP distance. Of the 40 isolates we choose to portray the 25 isolates with less than 100 SNPs difference. The isolates connected with the HCW at the surgical ward are highlighted and the isolate of the HCW in question is M5667. M6160 is a household contact. RH410 is the patient she nursed at the ward, who in turn was also positive for t267, and part of our outbreak cluster. M5746 is an unknown connection, but with no known link to the surgery ward. Between these four isolates there are four SNPs at the most.
