# Supplementary figures and images for: A Sporadic Four-Year Hospital Outbreak of a ST97-IVa MRSA With Half of the Patients First Identified in the Community
Source: Front Microbiol. 2018 Jul 10;9:1494. doi: 10.3389/fmicb.2018.01494 (PMC6048196; doi:10.3389/fmicb.2018.01494)

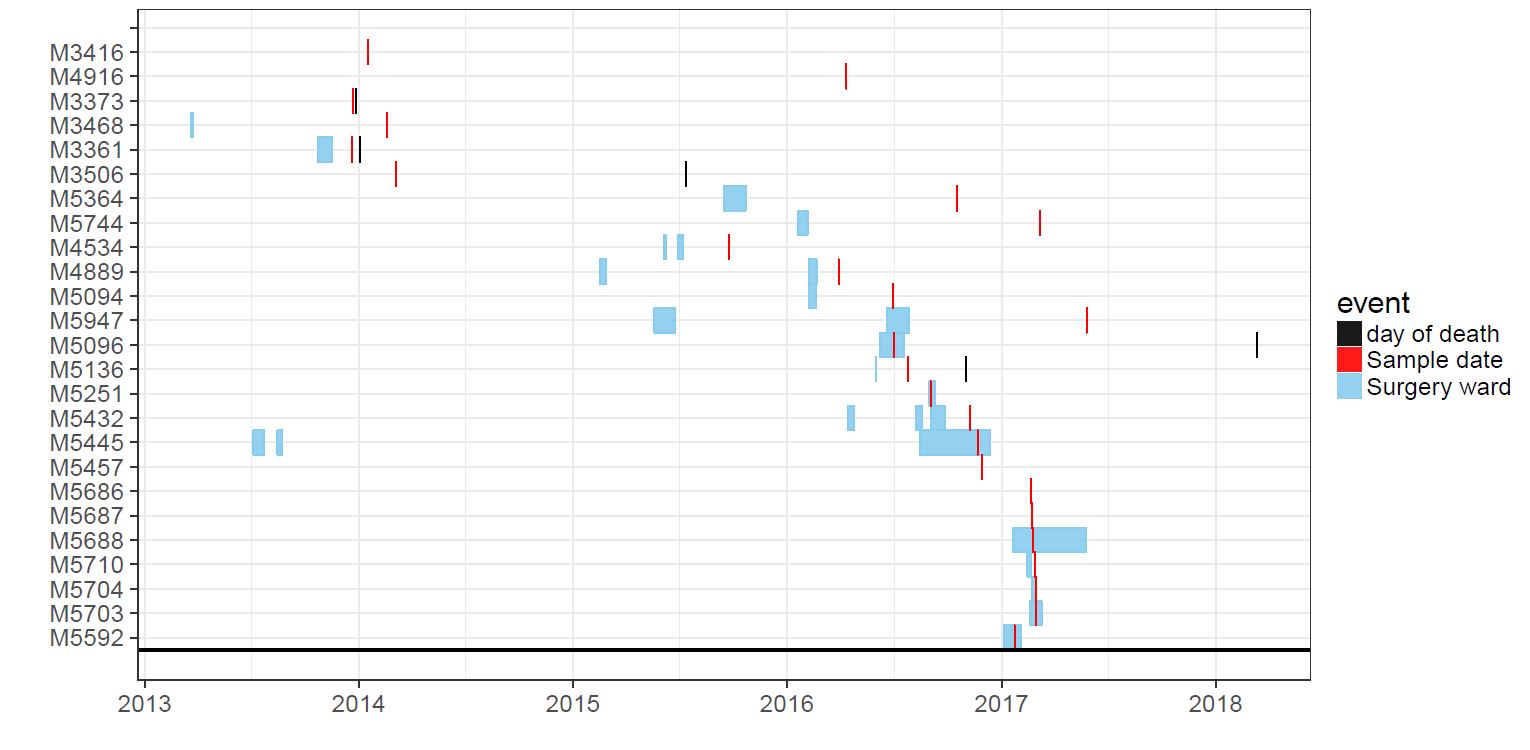

Supplement: FIGURE S1 — Timeline of hospitalization dates at the surgery ward and date of first positive MRSA sample. Thirteen patients had an overlap with at least one other patient. M5136 had an admission in June 2016, but the exact dates were not available. Sample date denotes the date when MRSA was first discovered in the patient. [file Image_1.JPEG]

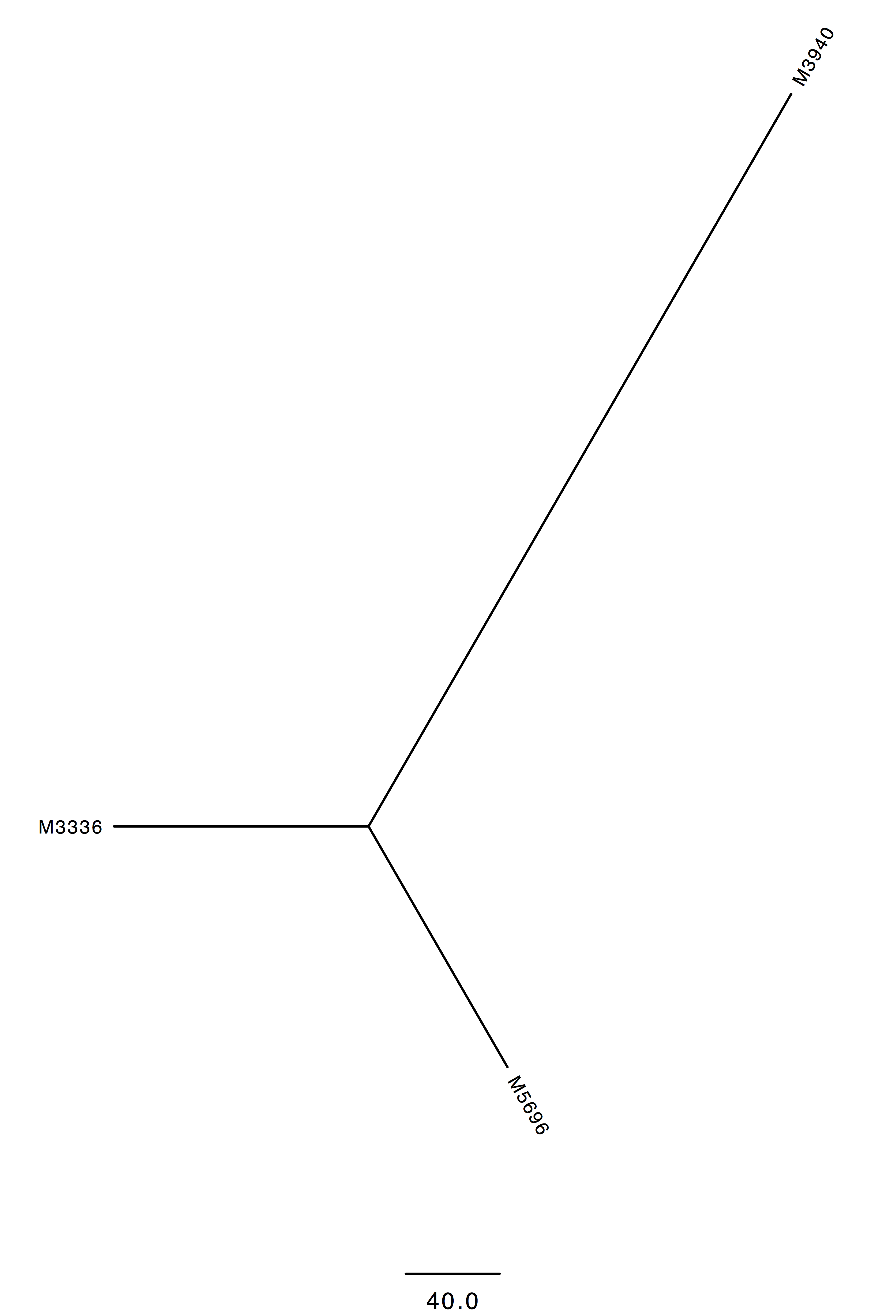

Supplement: FIGURE S2 — Neighbor-joining tree for the three isolates with t045/ST5-IVc/e. Scale bar indicates the SNP distance. M5696 is the HCW. The difference between the isolates was more than 200 SNPs. [file Image_2.JPEG]

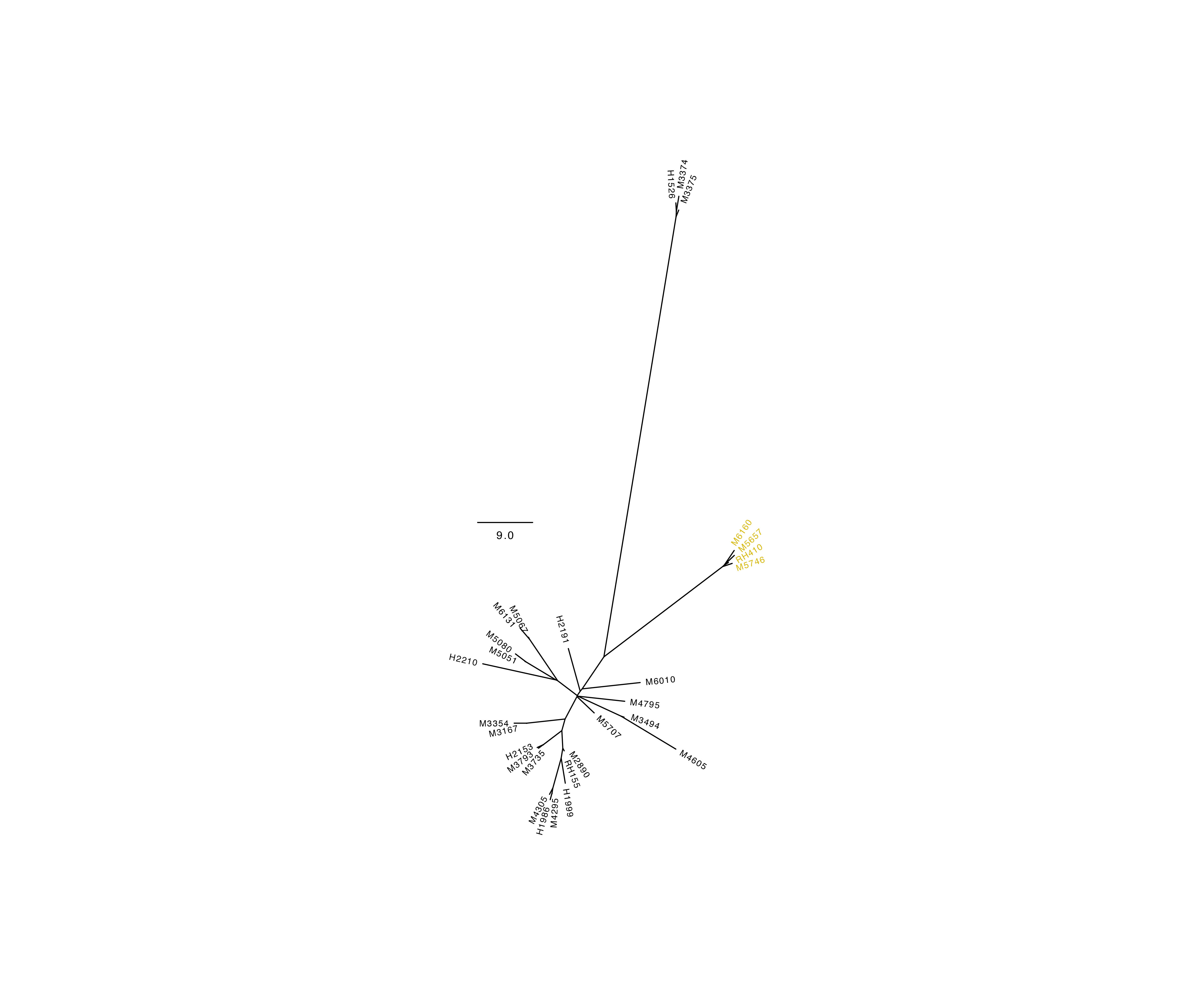

Supplement: FIGURE S3 — NJ-tree of the t002/ST5-IVg isolates. Scale bar indicates the SNP distance. Of the 40 isolates we choose to portray the 25 isolates with less than 100 SNPs difference. The isolates connected with the HCW at the surgical ward are highlighted and the isolate of the HCW in question is M5667. M6160 is a household contact. RH410 is the patient she nursed at the ward, who in turn was also positive for t267, and part of our outbreak cluster. M5746 is an unknown connection, but with no known link to the surgery ward. Between these four isolates there are four SNPs at the most. [file Image_3.JPEG]
